# Modeling the impact of ventilations on the capnogram in out-of-hospital cardiac arrest

**DOI:** 10.1371/journal.pone.0228395

**Published:** 2020-02-05

**Authors:** Jose Julio Gutiérrez, Jesus María Ruiz, Sofía Ruiz de Gauna, Digna María González-Otero, Mikel Leturiondo, James Knox Russell, Carlos Corcuera, Juan Francisco Urtusagasti, Mohamud Ramzan Daya

**Affiliations:** 1 Department of Communications Engineering, University of the Basque Country (UPV/EHU), Bilbao, Bizkaia, Spain; 2 Bexen Cardio, Ermua, Bizkaia, Spain; 3 Department of Emergency Medicine, Oregon Health & Science University (OHSU), Portland, Oregon, United States of America; 4 Emergentziak-Osakidetza, Basque Country Health System, Basque Country, Spain; Indiana University, UNITED STATES

## Abstract

**Aim:**

Current resuscitation guidelines recommend waveform capnography as an indirect indicator of perfusion during cardiopulmonary resuscitation (CPR). Chest compressions (CCs) and ventilations during CPR have opposing effects on the exhaled carbon dioxide (CO_2_) concentration, which need to be better characterized. The purpose of this study was to model the impact of ventilations in the exhaled CO_2_ measured from capnograms collected during out-of-hospital cardiac arrest (OHCA) resuscitation.

**Methods:**

We retrospectively analyzed OHCA monitor-defibrillator files with concurrent capnogram, compression depth, transthoracic impedance and ECG signals. Segments with CC pauses, two or more ventilations, and with no pulse-generating rhythm were selected. Thus, only ventilations should have caused the decrease in CO_2_ concentration. The variation in the exhaled CO_2_ concentration with each ventilation was modeled with an exponential decay function using non-linear-least-squares curve fitting.

**Results:**

Out of the original 1002 OHCA dataset (one per patient), 377 episodes had the required signals, and 196 segments from 96 patients met the inclusion criteria. Airway type was endotracheal tube in 64.8% of the segments, supraglottic King LT-D^™^ in 30.1%, and unknown in 5.1%. Median (IQR) decay factor of the exhaled CO_2_ concentration was 10.0% (7.8 − 12.9) with *R*^2^ = 0.98(0.95 − 0.99). Differences in decay factor with airway type were not statistically significant (*p* = 0.17). From these results, we propose a model for estimating the contribution of CCs to the end-tidal CO_2_ level between consecutive ventilations and for estimating the end-tidal CO_2_ variation as a function of ventilation rate.

**Conclusion:**

We have modeled the decrease in exhaled CO_2_ concentration with ventilations during chest compression pauses in CPR. This finding allowed us to hypothesize a mathematical model for explaining the effect of chest compressions on ETCO_2_ compensating for the influence of ventilation rate during CPR. However, further work is required to confirm the validity of this model during ongoing chest compressions.

## Introduction

As emphasized by current resuscitation guidelines, high quality cardiopulmonary resuscitation (CPR) is essential to improving outcomes of cardiac arrest victims [1]. CPR providers should deliver chest compressions of adequate depth (50–60 mm) with a rate of 100–120 compressions per minute (cpm). Observational studies have established that high quality chest compressions are associated with favorable outcomes [2–4]. However, the recommended values may not be optimal for all individuals [5]. Ideally, CPR should be guided based on patient’s response, e.g. using a non-invasive haemodynamic indicator [6, 7]. In this way, rescuers could adapt their CPR technique to optimize perfusion.

End-tidal carbon dioxide (ETCO_2_) is the partial pressure of carbon dioxide at the end of an exhaled breath. Experimental studies have shown that ETCO_2_ correlates with cardiac output and coronary perfusion pressure during CPR [8, 9]. Low ETCO_2_ values during resuscitation reflect the low cardiac output generated by chest compressions [10]. ETCO_2_ may serve as a non-invasive hemodynamic indicator, albeit with complexities of interpretation. A consensus statement published by the American Heart Association in 2013 recommended using the ETCO_2_ level as a physiological measure during CPR when an arterial or central venous catheter is not available [11]. Waveform capnography, i.e., continuous measurement of CO_2_ concentration with time, enables monitoring of ETCO_2_ during CPR. Current advanced life support (ALS) resuscitation guidelines [12, 13] emphasize the potential role of waveform capnography in monitoring CPR quality [14, 15], in the early recognition of return of spontaneous circulation (ROSC) during CPR [16, 17], and as a potential indicator of patient outcome [18–20].

During CPR, ETCO_2_ values depend on the blood flow generated by chest compressions, on ventilation rate and tidal volumes of each breath, and on the metabolic activity of the patient tissues [21, 22]. Chest compressions and ventilations have opposing effects on ETCO_2_ during CPR: compressions generate blood flow that delivers CO_2_ from the tissues to the lungs, with the amount of delivered CO_2_ being proportional to the amount of generated blood flow; ventilations, conversely, remove CO_2_ from the lungs, and thus ETCO_2_ decreases as ventilation rate is increased [23].

Recent studies have modeled the influence of chest compression quality (compression depth and rate) and ventilation rate on ETCO_2_ during CPR using multivariate analysis [14, 15]. Studies on ROSC detection and patient outcome rely on the comparison of measured ETCO_2_ levels [16–20, 24]. However, animal studies have suggested that ventilation rate significantly influences ETCO_2_ levels [25]. Consequently, when interpreting ETCO_2_ during CPR, ventilation rate acts as a significant confounding factor [22].

We hypothesized that the effect of ventilation on the capnogram could be modeled separately by analyzing variations in CO_2_ concentration during chest compression pauses. Modeling the impact of ventilation on the capnogram would have two main areas of application. First, it would facilitate a better assessment of the relationship between chest compression quality and capnography. Second, it would allow accounting for the confounding factor of ventilation rate in studies analyzing the correlation between ETCO_2_ and ROSC or patient outcome. In this context, the purpose of this study was to apply a novel strategy to model the impact of ventilations and ventilation rate on the exhaled CO_2_ measured in out-of-hospital cardiac arrest capnograms.

## Materials and methods

### Data collection

The data set used in this study was a subset of a large database of out-of-hospital cardiac arrest (OHCA) episodes collected from 2006 through 2016 by Tualatin Valley Fire & Rescue (TVF&R), an ALS first response emergency medical services (EMS) agency serving nine incorporated cities in Oregon, USA. The database is part of the Resuscitation Outcomes Consortium (ROC) Epidemiological Cardiac Arrest Registry collected by the Portland Regional Clinical Center. The data collection was approved by the Oregon Health & Science University (OHSU) Institutional Review Board (IRB00001736). Data were provided anonymous and contained no personal information.

Episodes were recorded using Heartstart MRx monitor-defibrillators (Philips, USA), equipped with capnography monitoring using sidestream technology (Microstream, Oridion Systems Ltd, Israel) and CPR quality monitors (Q-CPR). TVF&R used endotracheal tube or supraglottic (King LT-D^™^) devices to secure the airway. Ventilations were provided manually before and after patient intubation. For this study, we only included recordings with concurrent capnogram, compression depth signal, electrocardiogram (ECG) and transthoracic impedance (TI) signals.

### Segment selection

**Fig 1:**
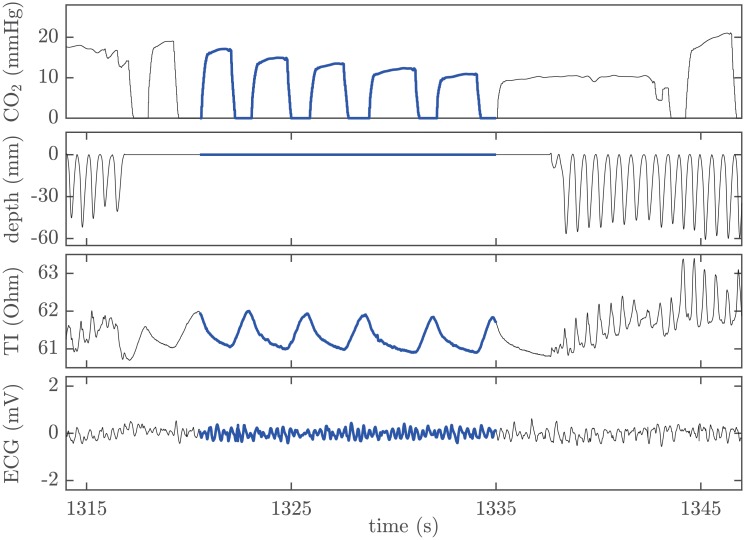
Example of segment selection. Required concurrent signals, with the segment highlighted in blue. From top to bottom: capnogram, compression depth, TI signal, and ECG.

Two biomedical experts (JJG and JMR) used a custom-made Matlab (Mathworks, USA) program to visually inspect the four signals extracted from each recording. Within each episode, they selected segments with no chest compressions where two or more complete ventilations were provided, and where the patient presented no spontaneous circulation. It was assumed that during those intervals, the decrease in the exhaled CO_2_ concentration was caused only by ventilations. Absence of chest compressions was verified using compression depth and TI signals. Ventilation instances were identified using both the capnogram and the TI signal. Absence of a pulse-generating rhythm was verified by inspecting the ECG. Pulseless electrical activity and perfusing rhythm were distinguished by checking the circulatory component of the TI signal [26]. The beginning of each segment meeting the inclusion criteria was annotated 3 seconds after the interruption of chest compressions, to reject the period where blood pressures change rapidly and blood flow induced by chest compressions decreases to a sustained low flow level [27–29]. Fig 1 shows an example of a selected segment, highlighted in blue. This segment presents five complete ventilations, which can be observed in the capnogram and in the TI signal as slow fluctuations. The flat line in the compression depth signal indicates the absence of chest compressions, confirmed by the cessation of the fast fluctuations caused by chest compressions in the TI signal. Finally, the artifact-free ECG segment reveals ventricular fibrillation.

### Data annotation

**Fig 2:**
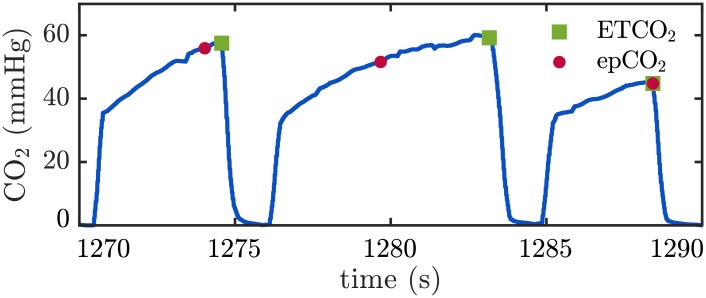
Example illustrating the metric epCO_2_. Short capnogram interval with three ventilations and the corresponding annotated ETCO_2_ (green squares) and epCO_2_ (red dots) values. The novel metric epCO_2_ was defined to represent the end-tidal values obtained if all ventilations had the same exhalation time. In the example, epCO_2_ value decays with each ventilation.

When analyzing the capnogram in the segments included in this study, we found that the duration of each ventilation cycle was different within each segment (see Fig 1). The duration of each ventilation affects the ETCO_2_ value since the plateau usually presents a low ascendant slope. To compare analogous points of exhaled CO_2_ pressure values in the ventilations of each segment, we annotated the CO_2_ value at a fixed delay from the beginning of the expiratory upstroke. We took the shortest plateau duration within each segment as the reference delay, and named this new metric *ensemble plateau* CO_2_ or epCO_2_. In the absence of spontaneous circulation, CO_2_ concentration during pauses in chest compressions decreases with each ventilation. However, this decrease may not be reliably measured since ETCO_2_ value is highly dependent on the duration of the expiratory plateau. Fig 2 illustrates this idea depicting a short capnogram interval with three ventilations and the corresponding annotated ETCO_2_ values (green squares). The value annotated in the second ventilation is higher than the value annotated in the first ventilation. The novel metric epCO_2_ was defined to represent the end-tidal values obtained if all ventilations had the same exhalation time. The values of epCO_2_ depicted with red dots in Fig 2, clearly show the expected decay with each ventilation.

**Fig 3:**
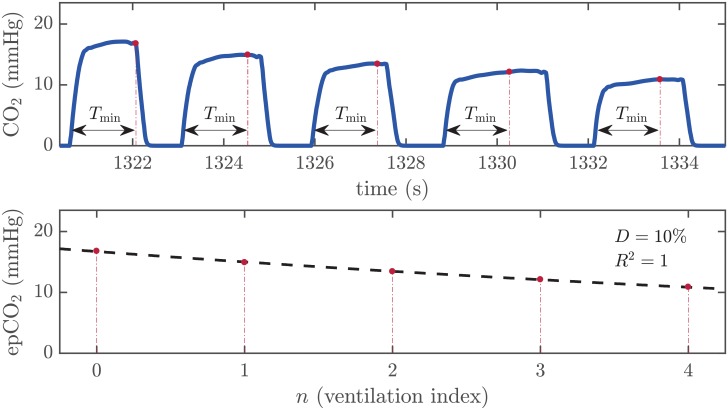
Data annotation and model fitting. Top panel: the annotated epCO_2_ values within the segment selected in Fig 1 are marked in the capnogram with red dots. Bottom panel: the epCO_2_ values are depicted as a function of the ventilation index, with the curve fitted using Eq (3) (dashed black line), and the decay factor and the coefficient of determination for the analyzed segment.

Top panel of Fig 3 shows the same capnogram segment from Fig 1 with the annotated epCO_2_ values depicted as red dots.

### Model fitting

The decay in epCO_2_, as illustrated in Fig 3, suggested an exponential decay model. Thus, we modeled the trend in epCO_2_ variation using the following expression:

$$\begin{array}{c} ep_{n}=k\cdot ep_{n-1}\;\text{for}\;n=1,2\ldots N-1, \end{array}$$ (1)

where *ep*_*n*_ represents the epCO_2_ value corresponding to the ventilation of index *n* within the segment and *N* the total number of ventilations in the segment. The decay factor between consecutive ventilations *D* (%) was computed as:

$$\begin{array}{c} D(\%)=100\cdot (1-k) \end{array}$$ (2)

Factor *k* was adjusted through a Matlab curve fitting tool using a decay exponential function as follows:

$$\begin{array}{c} ep_{n}=a\cdot b^{n}\;\text{for}\;n=0,1\ldots N-1 \end{array}$$ (3)

where *a* is an estimate of the initial epCO_2_ value (*n* = 0) in the segment and *b* provides the adjusted *k* factor for each segment. We used non-linear least squares as the fitting method.

Fig 3 illustrates the process of curve fitting for one segment. In the example, the fitting process yielded *a* = 16.7 mmHg and *b* = 0.90, indicating that on average epCO_2_ declined 10% with each ventilation.

### Statistical analysis

Values that did not follow a normal distribution according to the Lilliefors normality test were reported as median (IQR). Goodness of fit of the model was evaluated using the coefficient of determination *R*^2^, which provides a measure of the epCO_2_ variation that is explained by the model.

The distributions of the initial epCO_2_ values (annotated at the beginning of the segment), of the decay factor *D* and of *R*^2^ were represented using boxplots (a graphical depiction of the median, quartiles, and potential outliers). The relationship between *D* and the initial epCO_2_ value was analyzed using linear regression, and the coefficient of determination *R*^2^ was reported.

We also analyzed differences in the decay factor *D* with respect to the airway management technique (endotracheal or supraglottic). ANOVA analysis of variance was used to perform between groups comparisons since distributions were normal. P-values < 0.05 were considered significant.

## Results

The original database comprised 1002 distinct OHCA episodes. In 377 of them (37.6%) the four signals of interest (capnogram, compression depth, TI, and ECG) were concurrently available. After visual inspection, 196 segments from 96 episodes meeting the inclusion criteria were extracted for the study. Airway type was endotracheal tube in 64.8% of these segments and supraglottic in 30.1%. Airway type was unknown for 5.1% of the segments. The median ventilation rate measured in the included segments was 15.1 (10.5–20.9) ventilations per minute (vpm), much higher than the guidelines recommendation of 10 vpm after placement of an advanced airway.

**Fig 4:**
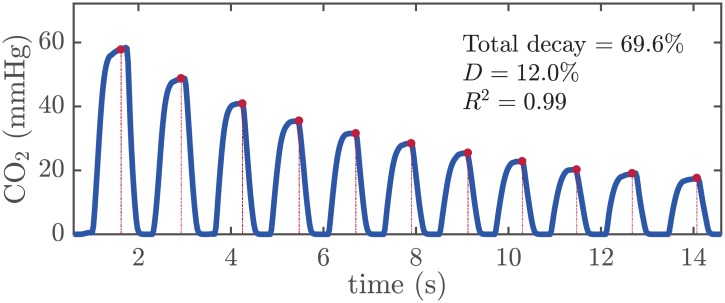
Example of epCO_2_ decay with ventilations during a pause in chest compressions.

Fig 4 shows an example segment. Eleven ventilations were provided to the patient during the chest compression pause. The model fitting results are depicted in the figure.

**Fig 5:**
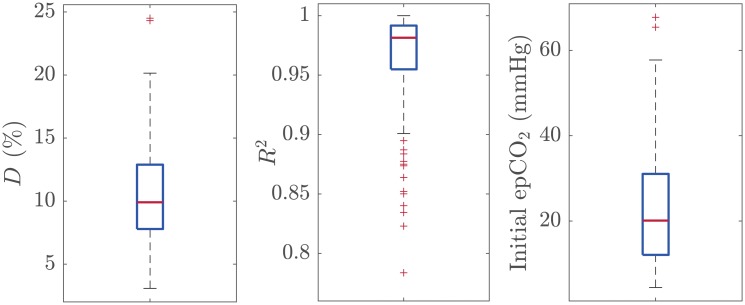
Distribution of the decay factor *D*, the coefficient *R*^2^ for the model, and the initial epCO_2_ value for all the segments included in the study.

Table 1 shows the median (IQR) of the decay factor *D*, of the coefficient *R*^2^ of the model fitted, and of the initial epCO_2_ value as a function of the number of ventilations in the segment (*N*). When all the segments were included, the median decay factor was 10.0% (7.8–12.9) with *R*^2^ equal to 0.98 (0.95–0.99), and the initial epCO_2_ value was 20.0 mmHg (11.9–31.0). Fig 5 shows the distributions of these three measures using boxplots.

**Table 1:** Number of segments (n), decay factor *D* (%), coefficient of determination *R*^2^ of the model, and initial epCO_2_ (ep_0_) in mmHg, as a function of the number of ventilations provided per segment (N). Values are reported as median (IQR).

	N = 2	N = 3	N = 4	N = 5	N >= 6	Total
Segments (n)	40	58	31	22	45	196
*D* (%)	12.0 (10.1–15.9)	10.3 (8.6–13.2)	8.9 (7.4–10.2)	9.6 (6.4–12.3)	8.8 (6.1–11.7)	10.0 (7.8–12.9)
*R*^2^	0.99 (0.96–1.00)	0.97 (0.96–0.99)	0.97 (0.94–0.99)	0.99 (0.96–0.99)	0.98 (0.96–0.99)	0.98 (0.95–0.99)
ep_0_ (mmHg)	21.0 (15.1–36.0)	20.7 (11.6–28.5)	21.9 (15.3–30.9)	17.2 (11.5–20.9)	22.7 (10.0–36.0)	20.0 (11.9–31.0)

Decay factor (*D*) was unrelated to initial epCO_2_ (*R*^2^ = 0.03).

We did not find significant differences in the decay factor with respect to the airway type, endotracheal or supraglottic (*p* = 0.17).

### Application of the findings

**Fig 6:**
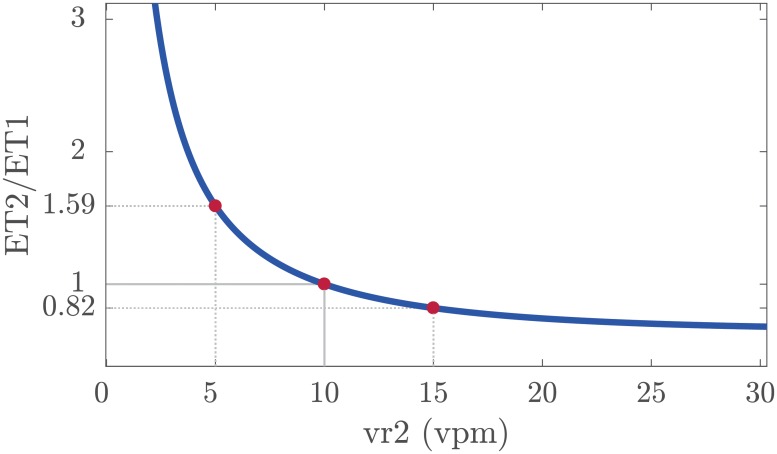
Graphical representation of the mathematical relationship of Eq (6) for a reference ventilation rate of 10 vpm and *k* = 0.9.

We have assessed the decrease in CO_2_ concentration caused by ventilations during chest compression pauses. Our results provide a potentially novel framework for the accurate interpretation of ETCO_2_ variation when chest compressions are provided. Our approach is based on the two hypotheses described below:

- First hypothesis: Contribution of chest compressions to the ETCO_2_ level between two consecutive ventilations.
  - Suppose that *ET*1 is the ETCO_2_ level after a given ventilation, and that *ET*2 is the ETCO_2_ level reached after the following ventilation when chest compressions are ongoing, as in intermittent ventilations after securing the airway.
  - In the absence of chest compressions, the ETCO_2_ level in the second ventilation would be *k* ⋅ *ET*1, in accordance to our model.
  - We propose that the contribution of chest compressions to the ETCO_2_ level between two consecutive ventilations could be estimated as *ET*2 − *k* ⋅ *ET*1.
  - Thus, that contribution per time unit could be expressed as:
    $$\begin{array}{c} \frac{ET2-k\cdot ET1}{t_{2}-t_{1}}, \end{array}$$ (4)
    where *t*_2_ − *t*_1_ is the duration of the ventilation.
- Second hypothesis: Variation of the ETCO_2_ level with respect to ventilation rate.
  - Suppose that the ETCO_2_ level keeps stable at *ET*1 mmHg during 1 minute of chest compressions when ventilation rate is *vr*1 vpm.
  - In the absence of chest compressions, the achieved ETCO_2_ level after *vr*1 ventilations would be *ET*1 ⋅ *k*^*vr*1^ according to our model.
  - We could then estimate the contribution of chest compressions to the ETCO_2_ level in that 1-min interval as:
    $$\begin{array}{c} ET1-ET1\cdot k^{vr1}=ET1\cdot \left(1-k^{vr1}\right) \end{array}$$ (5)
  - Now, for a different ventilation rate of *vr*2 vpm, and a stable ETCO_2_ level of *ET*2, the contribution of chest compressions would be *ET*2 ⋅ (1 − *k*^*vr*2^).
  - For chest compressions contributing equally to the ETCO_2_ level, we could write:
    $$\begin{array}{c} ET1\cdot (1-k^{vr1})=ET2\cdot (1-k^{vr2})\\ \frac{ET2}{ET1}=\frac{1-k^{vr1}}{1-k^{vr2}} \end{array}$$ (6)
    Taking the recommended ventilation rate of 10 vpm as the reference, i.e., *vr*1 = 10 vpm, Eq (6) expresses the ETCO_2_ level relationship as a function of ventilation rate. Fig 6 shows a graphical depiction of Eq (6) normalized to *vr*1 = 10 vpm, and for *k* = 0.9 (the median value reported in our results). For example, the ETCO_2_ level at a ventilation rate of 5 vpm would be 1.59 times the level at 10 vpm, chest compression performance being equal. Similarly, the estimated ETCO_2_ level at 15 vpm would be 0.82 times the ETCO_2_ at 10 vpm for the same compression performance. Thus, we could convert all ETCO_2_ measurements to a normalized ETCO_2_ value, by applying the corresponding correction factor (the inverse of the corresponding value in the vertical axis of Fig 6). Ultimately, we propose using this expression (6) for accurately comparing the ETCO_2_ level of different CPR intervals by correcting the confounding factor of ventilation rate.

## Discussion

We investigated the decrease in the exhaled CO_2_ concentration with each ventilation provided to the patient during CPR. Our aim was to isolate the effect of ventilations on CO_2_ concentration. This required that we identify pauses in chest compressions to select our analysis segments. Another advantage of this selection is that we had reliable capnogram tracings, since there is no presence of artifact caused by chest compressions compromising the analysis of ventilations [30, 31]. As the EMS agency that collected these episodes achieved very high chest compression fractions (minimal pauses), we could only identify 197 segments from the 1002 recordings.

When there is no CO_2_ exchange in the lungs, ventilation adds oxygen to highly CO_2_ concentrated volumes, so that CO_2_ concentration decreases (CO_2_ dilutes) in the lungs. The new concentration depends on the functional residual capacity, on the patient anatomic dead space and on the ventilation volume. Since we analyzed consecutive ventilations in each segment, similar volumes were assumed. For this reason, our model fitted the data very well, as proven by the high *R*^2^ obtained (Fig 5, middle panel). However, the estimated decay factor *D* per segment showed a moderate dispersion (Fig 5, left panel) with a median (IQR) of 10% (7.8–12.9). This dispersion could be attributable to differences in patient functional residual capacity and anatomic dead space, and in the ventilation volumes provided with each breath. Since those parameters are unknown in the field, the median decay factor of 10% per ventilation may be useful as a sensible reference level. For example, in the absence of perfusion replenishing CO_2_, the median decrease after 10 ventilations could be estimated as 65.1% (i.e, 100 ⋅ (1 − 0.9^10^)) of the initial epCO_2_ value. Fig 4 shows a segment of our database in which 10 ventilations were administered after the initial epCO_2_ value. In this case, the actual decrease in epCO_2_ after 10 ventilations was 69.6%, very close to the estimated value (1.59 and 0.82, respectively).

The decay factor *D* had no correlation with the initial epCO_2_ value (*R*^2^ = 0.03), i.e., the initial epCO_2_ predicted almost none of the variation in the decay factor, favoring the strength of the model. This finding shows that the decay factor is not related to the factors that condition the initial epCO_2_ value of each segment, such as the cardiac arrest etiology, the initial rhythm, the airway type, or the chest compression quality (rate, depth, recoil) and the ventilation rate being administered before the analyzed segment. We found a considerable number of segments (13.8%, 27/196) with initial epCO_2_ values higher than 40 mmHg (Fig 5, right panel). Most of these segments corresponded to the beginning of the capnogram in the file, when CO_2_ concentrations different from zero started to be measured. A possible explanation is that these episodes could correspond to primary respiratory failure leading to cardiac arrest [32], although we were unable to confirm this since we did not have any clinical data regarding etiology of the cardiac arrest. Other segments with high epCO_2_ values were linked to low ventilation rates in the previous minute (between 2 and 4 vpm).

Our study has allowed us to model the ETCO_2_ variation with ventilation rate during CPR. In a swine model of cardiac arrest, Gazmuri et al. used mechanical ventilation controlling tidal volume and respiratory rate [25]. Authors adjusted a curve to their experimental data which provided the variation of ETCO_2_ level as a function of the ventilation exchanged volume in time (measured in liters per minute). Considering an average swine weight of 33 kg and a constant tidal volume of 6 ml/kg per ventilation, the ETCO_2_ level at a constant ventilation rate of 5 vpm was 1.75 times the ETCO_2_ level at 10 vpm. The ETCO_2_ at 15 vpm was 0.75 times the level at 10 vpm. Their curve was similar to the one we have depicted in Fig 6, and reported values by Gazmuri et al. were comparable to those obtained applying our hypothesis.

One of the potential clinical applications of our study is to facilitate the analysis of the relationship between ETCO_2_ and CPR quality. Resuscitation guidelines encourage the use of waveform capnography to monitor CPR quality. However, the variation of ETCO_2_ with ventilation rate and chest compression quality parameters is not yet well understood, and current guidelines do not establish any specific ETCO_2_ target to provide guidance on CPR quality.

Two recent studies have investigated these relationships [14, 15]. Sheak et al. conducted a multicenter cohort study of 583 in-hospital and out-of-hospital cardiac arrests. After averaging ETCO_2_ values, compression depth, compression rate and ventilation rate over 15-s epochs, they used a multiple linear regression model to predict ETCO_2_ variation based on the other three variables [14]. In their study, for every 10 mm increase in depth, ETCO_2_ rose 1.4 mmHg (*p* < 0.001); for every 10 vpm increase in ventilation rate, ETCO_2_ dropped 3.0 mmHg (*p* < 0.001); and compression rate was not a predictor of ETCO_2_ variation. Murphy et al. conducted an observational prospective study with similar objectives including 230 patients [15]. In this case, ETCO_2_ level, chest compression data and ventilation rate were averaged over 1-min epochs. The association between log-transformed ETCO_2_ and CPR variables was assessed through linear mixed effect models. The authors concluded that a 10 mm increase in compression depth was associated with a 4.0% increase in ETCO_2_ (*p* < 0.0001); a 10 vpm increase in ventilation rate with a 17.4% decrease in ETCO_2_ (*p* < 0.0001); and a 10 cpm increase in compression rate with a 1.7% increase in ETCO_2_ (*p* = 0.02) [15].

Comparison of the studies is challenging because Sheak et al. reported absolute differences, while Murphy et al. reported relative differences. In any case, their results are quite distinct. Estimated variations with compression depth in both studies would only match for an average ETCO_2_ level of 35 mmHg, and variations with ventilation rate only for an average ETCO_2_ level of 17.2 mmHg. Additionally, the conclusions of both studies significantly diverge from what would be expected during resuscitation episodes. According to their results, increasing compression depth from 30 to 50 mm (from suboptimal to the minimum recommended depth) would only raise ETCO_2_ by 2.8 mmHg (or 8%). The main factor compromising the applicability of their models is that the nature of dependence of ETCO_2_ variations with compression variables and with ventilation rate may differ, i.e., it may not be linear or logarithmic for all the studied variables. Including all of them in the same model may have an additional confounding effect. According to the novel approach presented in our study, the change in ETCO_2_ in a given interval which is attributable to chest compressions could be estimated by removing the influence of concurrent ventilations, which we can now model.

Another clinical application of our findings is related to the interpretation of ETCO_2_ as an indicator of ROSC and prognostication during CPR. Resuscitation guidelines highlight that an increase of ETCO_2_ during CPR may indicate ROSC, and that low ETCO_2_ values may reflect a poor patient prognosis. However, studies in this field have not yet achieved high sensitivity and specificity in ROSC detection, nor reported a strong correlation between the ETCO_2_ level and resuscitation outcome [16–20, 24]. We suggest that, since the ETCO_2_ level varies significantly with ventilation rate, this parameter may act as an important confounding factor in the cited studies. Our results provide a way of compensating for the effect of ventilation rate when analyzing ETCO_2_ values.

### Limitations

There are several limitations in our study that could be grouped in two categories. The first category refers to the proper definition of the model during pauses in chest compressions, and the other one to the extrapolation of the model to the scenario of ventilations during ongoing chest compressions.

The mathematical model for the epCO_2_ decay with each ventilation has been obtained under the assumption of equal tidal volume per ventilation. Although ventilations are consecutive within each segment, there is no evidence that consecutive ventilations are administered with a similar tidal volume. The value of ETCO_2_ depends on the ventilation rate and tidal volume provided with each ventilation [25]. Absence of volume data, a common situation in pre-hospital settings is an important limiting factor of the model.

Considerably different duration of plateau phases found in our capnograms compelled us to rely on a modified measure of ETCO_2_ for a systematic comparison of consecutive ventilations. This does not correspond to how ETCO_2_ is formally measured (at the end of the plateau phase), so we defined a new metric: epCO_2_. This metric attempts to estimate the value of ETCO_2_ that would have been measured if all ventilations in the compression pause would have had the same duration. This approximation may introduce an error in the model.

Approximately 75% of the ventilation rates measured in our segments were above the recommended 10 vpm. Our data are consistent with previous works reporting that excessive ventilation rates are common in resuscitation [33, 34]. Hyperventilation may result in significantly increased intrathoracic pressure and decreased coronary perfusion pressures and survival rates.

We applied a 3-s guard to annotate the beginning of each segment, considering that a sustained low flow state is reached after that delay from the interruption of chest compressions. This time may not be generalized for every patient.

When the model is extrapolated to the ongoing chest compressions scenario we only took into account the influence of ventilation rate on the variation of ETCO_2_. We did not considered factors associated to chest compressions that have a role in ETCO_2_ variation, such as the following:

Chest compressions with a depth compliant with current guidelines produce measurable and substantial ventilation volumes [35], with 81% of the passive tidal volumes recorded during chest compressions being lower than 20 ml. Chest compressions alone do not provide physiologically significant tidal volumes but may produce alterations in the capnogram.

During compressions, intrathoracic pressure increases and the lung volume decreases [36]. Lung volume becomes lower than the functional residual capacity, which is recovered only when CPR is interrupted. Lung volume reduction during chest compressions can promote progressive atelectasis and pulmonary congestion. These interactions caused by chest compressions may involve alterations of the ETCO_2_ level, not contemplated in our model.

Intrathoracic airway closure is a phenomenon associated with lung volume reduction limiting the delivered ventilation [36]. The negative pressure produced by chest compressions in the alveoli cannot be transmitted at airway opening and no inspiratory flow is generated. No respiratory tidal volume can be produced during decompression affecting the exhaled CO_2_.

Studies related to ventilation during CPR are scarce. The complex relations between compressions and ventilations during CPR, although still not well understood, could somehow modulate the exhaled CO_2_ concentration [33, 36].

We are aware of the simplicity of the proposed model and of the role of the confounding factors that affect ETCO_2_ values during resuscitation besides ventilation rate, such as the etiology of the cardiac arrest or the administration of drugs [22], which were unavailable in our retrospective data. These other considerations may be unknown during treatment as well, necessitating reliance on the limited data available in real time. Our method may overcome one of the many confounders of ETCO_2_ interpretation during chest compressions. The formulae proposed in the present study are promising hypotheses, but need to be confirmed with further analysis of resuscitation recordings.

## Conclusion

We have modeled the decrease in exhaled CO_2_ concentration with ventilations during chest compression pauses in CPR. On average, each ventilation produced a decrease of 10% in the measured exhaled CO_2_ value. This finding allowed us to hypothesize a mathematical model for explaining the effect of chest compressions on ETCO_2_ compensating for the influence of ventilation rate during CPR. However, further work is required to confirm the validity of this model during ongoing chest compressions.

## Supporting information

S1 File Annotations and curve fitting results for all segments included in the study. (XLSX)
